# Acceptability of screening for substance use among children at Ugandan health facilities

**DOI:** 10.1186/s13011-026-00716-7

**Published:** 2026-03-03

**Authors:** Harriet Aber-Odonga, Juliet Ndimwibo Babirye, David Musoke, Nazarius Mbona Tumwesigye, Ingunn Marie S. Engebretsen, Fred Nuwaha

**Affiliations:** 1 School of Public Health, Makerere University College of Health Sciences, P.O Box 7072, Kampala, Uganda; 2 Centre for International Health, Department of Global Public Health and Primary Care, University of Bergen, Bergen, Norway

**Keywords:** Acceptability, CRAFFT, Screening, Alcohol, Substance use disorders, Child, Adolescent, Caregiver, Health worker, Health facilities, Uganda

## Abstract

**Background:**

Substance use among children and adolescents is an emerging public health concern, with studies from some settings reporting substantial levels of alcohol and other substance use among young people. However, in many low- and middle-income countries, including Uganda, the role, feasibility, and implications of routine substance use screening in primary healthcare settings remain poorly understood. Validated tools such as the CRAFFT (Car, Relax, Alone, Forget, Friends, Trouble) screening instrument have been used in other contexts, but evidence on their acceptability among children, caregivers, and health workers in African primary care settings is limited. This study assessed the acceptability of the CRAFFT tool among children aged 6–17 years, their caregivers, and health workers in primary healthcare settings in Uganda.

**Methods:**

A cross-sectional study was conducted with 824 children, their caregivers, and 47 health workers across selected health facilities. Acceptability was measured using Likert-scale questionnaires tailored to each participant group; children, caregivers, and health workers alongside open-ended questions. Quantitative data were analyzed using STATA 18, and qualitative feedback was analyzed thematically.

**Results:**

The CRAFFT screening tool was generally acceptable across all participant groups. Children found the tool easy to understand and informative, caregivers appreciated its potential for early identification of risky behaviors, and health workers viewed it as useful in identification of substance use. However, several concerns were raised, including long screening durations, lack of privacy, cultural and religious sensitivities, and doubts about the relevance of screening younger children.

**Conclusion:**

CRAFFT screening was found to be acceptable among children, caregivers, and health workers in Ugandan health facilities. However, successful implementation requires addressing key barriers such as confidentiality, time burden, and cultural appropriateness. Integrating screening into routine care, especially in community and school settings, may enhance uptake and early intervention for substance use among children and adolescents.

## Background

Growing evidence indicates that early initiation of alcohol and other substance use among children and adolescents is an important global public health concern, although it remains under-recognized and under-addressed in many health systems [1]. In sub-Saharan Africa, alcohol use among children under 10 years has been estimated at up to 10% in some settings [2], highlighting the need for earlier identification and appropriate responses. Despite this, substance use among younger populations frequently goes undetected in primary healthcare (PHC) settings, where routine screening and developmentally appropriate interventions for children and adolescents are uncommon.

Substance use screening, often delivered as part of Screening, Brief Intervention, and Referral to Treatment (SBIRT), has been widely promoted in high-income countries as a strategy to identify risky substance use and initiate early support. In adult populations, particularly for alcohol use, systematic reviews have shown that screening combined with brief interventions can reduce risky drinking behaviors [3, 4]. Among adolescents, SBIRT has been shown to be feasible and acceptable in pediatric primary care and school-based settings, with modest reductions in alcohol and cannabis use reported in some trials [5–7]. However, the strength of this evidence is mixed, and effects are often small to moderate, highly context-dependent, and shaped by developmental stage, confidentiality concerns, provider capacity, and health system readiness.

In line with this evidence, several professional bodies in high-income settings recommend routine or universal screening. The Substance Abuse and Mental Health Services Administration (SAMHSA) and the American Academy of Pediatrics recommend universal substance use screening for adolescents as part of routine health visits [8]. Similarly, the U.S. Preventive Services Task Force (USPSTF) recommends screening adults aged 18 years and older for unhealthy drug use using standardized questions, provided that adequate diagnostic, treatment, and referral services are available [9]. However, the USPSTF concluded that evidence is insufficient to determine the balance of benefits and harms of screening for unhealthy drug use among adolescents, even in well-resourced health systems. These recommendations highlight that screening is not inherently beneficial in all contexts and that its effectiveness depends on the availability of follow-up care, trained providers, referral pathways, and safeguards against potential harms such as stigma, mislabeling, or opportunity costs for healthcare workers [10].

Primary healthcare is often the first point of contact with the health system for children and adolescents, including those who use substances, who may present for unrelated health concerns [11]. In theory, integrating substance use screening into PHC could offer opportunities for early identification and support [12]. In practice, however, especially in low- and middle-income countries (LMICs), evidence on the clinical effectiveness, cost-effectiveness, and feasibility of routine screening in PHC remains limited [13–15]. Implementation challenges including high patient volumes, limited time, insufficient training, weak referral systems, and competing health priorities raise important questions about whether and how screening should be implemented in such settings [16–18].

In Uganda, the Ministry of Health has recommended screening for alcohol and substance use within PHC, but implementation remains inconsistent and largely focused on select populations, such as pregnant women and individuals receiving HIV care where patients are screened for alcohol and drug use without standard screening tools [19]. A national manual for Screening, Brief Intervention, and Referral to Treatment for alcohol use disorder has been drafted but is not yet finalized [20]. At the same time, emerging evidence suggests substantial levels of substance use among children, including those aged 5–8 years, with prevalence estimates ranging from 7 to 8% using diagnostic tools such as the MINI-KID, and up to 25% when screened using the CRAFFT tool in school settings [21, 22]. Despite these findings, routine screening for children and adolescents is rarely conducted in PHC, and psychosocial support is often deferred to higher-level facilities. Within Uganda’s stepped-care model, PHC facilities are expected to manage harmful substance use and common mental health conditions through basic counselling and psychosocial support, with referral of more severe cases to district or national referral hospitals for specialized care [23]. In practice, this care cascade is frequently undermined by limited human resources, time constraints, and the absence of standardized, developmentally appropriate screening tools for younger populations [23, 24]. These constraints raise concerns about the feasibility, acceptability, and potential unintended consequences of introducing routine screening without adequate system support.

Although tools such as the CRAFFT screening instrument have been validated against the MINI-kid in Uganda among children aged 6–13 years and other settings [25], evidence on their acceptability for routine use among younger children, caregivers, and health workers in LMIC primary care settings remains scarce. Most existing studies on CRAFFT acceptability focus on adolescents aged 12 years and older, despite evidence that substance use may begin at much younger ages [21]. Understanding acceptability across different stakeholder groups is therefore a critical first step before considering broader implementation.

Against this backdrop, this study assessed the acceptability of the CRAFFT screening tool among children aged 6–17 years, their caregivers, and health workers in primary healthcare settings in Uganda.

## Methods

### Study design

This was a cross-sectional health facility study conducted in the outpatient departments among children, their caregivers, and health workers between June and September 2022 in Mbale Uganda. Detailed methods of the study have been published [26], and below follows a summary:

### Study setting

Mbale which is administratively sub-divided into the City and District has 54 health facilities, two private hospitals, and one regional referral hospital serving the eastern region. The population in Mbale was estimated at 590,000 in 2020 [27].

### Study participants

Study participants included children aged 6–17 years who attended the outpatient departments for other medical conditions along with their caregivers.

At each facility, one health worker from the outpatient department was also invited to participate.

### Sample size and sampling procedure for child and caregiver populations

A sample size of 854 child or adolescent-caregiver pairs was estimated using the Kish Leslie (1965) formula as described by Aber-Odonga et al. [26]. We used a two-stage sampling procedure to identify study participants. In the first stage, all (57) general healthcare facilities in Mbale district were considered for inclusion, while specialized clinics such as women’s and HIV clinics were excluded. This exclusion was intentional, as these specialized facilities routinely screen for alcohol and substance use, unlike general health facilities where such practices are less common. Including them could have introduced bias by overrepresenting better-performing facilities, thus compromising the study’s objective to assess typical service delivery in general healthcare settings. In the second stage, a probability proportionate to size approach was used to estimate the sample size for each selected facility based on patient attendance at each facility. At the second stage, consecutive eligible patients seeking care for various medical reasons were approached for study inclusion until the sample size for that facility was obtained.

The selection of the health worker participating in this study was done using computer generated random numbers.

### Data collection

Data for this study were collected using a pretested semi-structured questionnaire. Face-to-face interviews were conducted with children first, followed by their caregivers, using hard-copy questionnaires. Health workers completed a self-administered questionnaire. The pretest was done at a Level IV health facility in Kampala to identify and resolve any ambiguities and ensure clarity of questions. The tools included both structured and open-ended questions on acceptability. Trained health workers conducted the interviews with children, adolescents and caregivers and children.

#### Piloting of the CRAFFT tool

In this study, selected health workers were trained to use the CRAFFT tool for screening for alcohol and other substance use. They participated in a three-day training that covered the use of the CRAFFT tool, providing brief advice to children with a positive screen, and understanding of the referral processes. The training also included instructions on recording responses and collecting data for this study. The CRAFFT screening tool was chosen for this study because it is suitable for screening children and adolescents [28]. The translation, adaptation and psychometric evaluation of the CRAFFT tool in our setting has been published [25]. As part of the validation process, the CRAFFT screening tool was translated into *Lumasaaba* the predominant local language spoken in Mbale and adapted to fit the cultural and contextual realities of the study setting. The *Lumasaaba* version demonstrated strong psychometric properties, including high internal consistency (Cronbach’s α = 0.86) and strong inter-item reliability (Spearman correlation coefficient = 0.84). Additionally, it showed excellent sensitivity (91%) and specificity (92%).

The version used in this study was the contextually adapted CRAFFT tool. The questions presented in *italics* below are the modified items from the adapted version:

1. Have you ever driven/been driven by someone using a CAR/bicycle/motorcycle/scooter or bodaboda while you/they were drunk, or high or had been using alcohol/drugs?
2. Do you ever use alcohol/drugs (marijuana, tobacco) to RELAX, feel better about yourself/ be able to sleep/perform better or fit in (Not to feel shy/be accepted/ fit in group/ be same as others?
3. Do you ever use alcohol/drugs while you are by yourself, or ALONE (when nobody is seeing you)?
4. Do you ever FORGET (not remember) things you did when you had drunk alcohol/ used other drugs?
5. Do your FAMILY (parents, brothers, sisters, relatives, or other people who stay in your home) or FRIENDS ever tell you that you should reduce/stop drinking/use of other drugs?
6. Have you ever gotten into TROUBLE while you were using alcohol or other drugs?

### Primary outcome

#### Measurement of acceptability

We adapted the Theoretical Framework of Acceptability (TFA) to guide questionnaire development for acceptability [29]. Table 1 illustrates the adaptation of the constructs from the framework to measure acceptability in this study.

**Table 1:** Adaptation of questionnaire items to the theoretical framework of acceptability (TFA) constructs

TFA Construct	Definition	Corresponding Questionnaire Items
Affective Attitude	How an individual feels about the intervention (positive or negative emotions)	- Satisfaction with the explanation for participation in the study - Comfort level in answering the CRAFFT questions
Burden	The perceived amount of effort required to participate	- Ease of answering the CRAFFT questions - The time taken to complete the CRAFFT tool
Ethicality	The extent to which the intervention fits with the individual’s values	- Satisfaction with screening being conducted during care for other health conditions - Satisfaction with screening being conducted by a health worker
Intervention Coherence	The extent to which participants understand the intervention and how it works	- Satisfaction with the explanation for participation in the study - Experience of being asked about substance use
Opportunity Costs	The extent to which benefits or values must be sacrificed to participate	*(Not explicitly covered in the listed items)*
Perceived Effectiveness	The extent to which the intervention is perceived as likely to achieve purpose	- Likelihood of encouraging family and friends to be screened using the CRAFFT tool
Self-efficacy	Confidence that one can perform the behaviors required by the intervention	- Willingness to answer substance use questions at another time if recommended by a health worker

Acceptability of the CRAFFT tool among children or adolescents was assessed using a nine-item questionnaire while caregiver acceptability was assessed on an 8-item questionnaire. The questions evaluated satisfaction with the explanation for participation in the study, ease of answering the CRAFFT questions, comfort level in answering the questions, the time taken to complete the CRAFFT tool, willingness to answer substance use questions at another time if recommended by a health worker, likelihood of encouraging family and friends to be screened using the CRAFFT tool, their experiences with being asked about substance use, satisfaction with the screening being conducted during care for other health conditions, and satisfaction with the screening being conducted by a health worker. Responses were rated on a scale from 1 (very dissatisfied/uncomfortable) to 5 (very satisfied/comfortable), with additional options ranging from 1 (too long) to 3 (acceptable time). Additionally, open ended verbal comments from children and caregivers regarding the instrument’s acceptability were documented by health workers.

#### Health worker acceptability

Health worker acceptability of the CRAFFT screening was assessed using a seven-item questionnaire. The first seven questions evaluated the tool’s effectiveness in helping clinicians understand patient problems, helping patients feel understood, its usefulness in screening for alcohol use, screening for substance use, for use as a referral tool, in developing treatment plans and the ease of incorporating it into patient care. These questions were scored on a five-point Likert scale from 1 (Not at all) to 5 (Very). The final question was open-ended, asking health workers to document their experiences, challenges, and recommendations regarding the administration of the CRAFFT tool.

Additional data was collected on the sociodemographic characteristics of the patients, including age, gender, and education level. For the caregiver additional data included age, gender, marital status, educational level, religion, household size, polygamy, and violence at home were collected. Health worker characteristics such as age, gender, position at the health facility, and work experience, were also collected.

### Data management and analysis

During data collection, AOH reviewed all completed questionnaires daily to identify any errors or missing data. If any gaps were found, the respective enumerator was contacted and reminded of the correct data collection procedures. For data entry, we used Excel spreadsheets with double data entry by two independent data entrants cross-referencing each other’s work to enhance completeness. Any discrepancy between the two datasets was resolved by AOH who referred to the original hard copy questionnaires to achieve this.

Data in Excel was exported into STATA software version 18 for further cleaning and statistical analysis. To summarize the data, we calculated means and standard deviations for continuous characteristics, and frequencies and proportions for categorical socio-demographic characteristics, acceptability, and CRAFFT scores. For the open-ended questions, feedback from children, caregivers, and health workers was reviewed, coded, and analyzed to generate frequencies and proportions. We analyzed the verbal feedback from participants using thematic content analysis. Responses were transcribed and reviewed by two researchers who independently generated initial codes based on recurring sentiments. These codes were then grouped into broader themes of positive and negative feedback. Frequencies and percentages were calculated for each theme.

### Ethical consideration

The project within which this study was conducted obtained ethical approval from the Regional Committees for Medical Research Ethics-South East Norway with reference number 50,146. Additionally, the study received independent ethical approval from the Makerere University School of Public Health Higher Degrees Research and Ethics Committee (SPH-2022-224) and the Uganda National Council for Science and Technology (HS2182ES). To uphold participants’ rights and ensure their voluntary participation, a parent/legal guardian gave informed consent for their own participation as well as the participation of their children or adolescents in this study including children who were illiterate. Additionally, after obtaining parental/legal guardian informed consent for the child or adolescent to participate in the study, children or adolescents were asked to provide assent to participate in the study. To ensure ethical conduct while working with this vulnerable population, several measures were implemented. Parents and caregivers were first asked to provide permission for their children to participate in the study. As part of this process, they were also asked to allow the child to speak privately with a trained health worker during the screening interview.

Children identified with *mild substance use* received brief advice and counselling immediately on-site by the health worker following the interview. For those who screened positive for *probable substance use disorders*, referral support was provided through a designated contact at the Regional Referral Hospital, where they could access more specialized mental health and substance use services. While we did not provide direct treatment as part of the study, we facilitated linkage to appropriate care within the existing health system.

No financial compensation was offered to either children or caregivers for their participation. This decision was made to avoid coercion and ensure voluntary participation. However, all participants were informed of the study’s purpose, their right to withdraw at any point, and the available support services for those in need.

Some respondents under 18 years of age gave consent and were interviewed without a caregiver since they were considered emancipated minors due to the fact that they were married. The research adhered to GCP-ICH guidelines.

## Results

### Socio demographic characteristics of children, caregivers and health workers

Of the 854 children who responded to the CRAFFT tool, 834 (97.7%) completed the screening as we excluded 20 missing records. In this screening exercise, 27.8% (232/834) of the participants met the criteria for either probable alcohol use disorder (AUD) or probable substance use disorder (SUD). Specifically, 25.3% (211/834) screened positive for probable AUD, while 3.8% (32/834) met the criteria for probable SUD. The average time required to complete the CRAFFT screening test was 18.7 min (SD = 16.3) for those with probable SUD and 17.7 min (SD = 15.7) for those without. More than half of the participating children, 430 (51.7%), were aged between 14 and 17 years, and the majority 610 (73.1%) had attained primary-level education. Among the participating caregivers, more than two-thirds, 561 (69.3%), were female, and more than half 440 (54.2%) were farmers. (Table 2)

**Table 2:** Characteristics of children (*N* = 824) and their caregivers (*N* = 808)

Variables	Frequency	Percent
**Child age (years)**		
6–9	157	18.9
10–13	245	29.5
14–17	430	51.7
**Sex**		
Male	420	50.9
Female	404	49.1
**Highest level of Education**		
None/ primary	610	73.1
Secondary	224	26.9
**Caregiver characteristics**		
**Age**		
14–29	141	17.5
30–39	255	31.6
40–49	271	33.5
50+	140	17.4
**Sex**		
Male	248	30.7
Female	561	69.3
**Education level**		
None	70	8.7
Primary	329	40.7
Secondary	231	28.6
Tertiary	178	22.0
**Religious affiliation of caregiver**		
Protestant	261	32.3
Catholic	199	24.6
Pentecostal	141	17.5
Seventh Day Adventists	47	5.82
Muslim	160	19.8
**Caregiver occupation**		
Vendor	128	15.7
Farmer	440	54.2
Salaried worker	244	30.1
**Caregiver Marital status**		
Cohabiting	101	12.6
Married	532	66.4
Divorced/separated	107	13.4
Widow	61	7.6
**Tribe**		
Lumasaaba	585	72.2
Others	225	27.8
**Household size**		
1–4	192	24.0
5–8	487	60.8
9–10+	122	15.2

### Health worker characteristics

A total of 47 of health workers from the 57 units participated in the study giving a response rate of 82.5%. Specifically; 34 health workers from HC III facilities, 8 from HC II facilities, 3 from HC IV facilities, and 2 from the Regional Referral Hospital. All health workers had attained tertiary education. Approximately half 24 (51.1%) were under the age of 29, with a mean age of 31.9 years (SD = 8.4). Additionally, more than half 28 (59.6%) were female. The average clinical experience was 6 years (SD = 5.5). Furthermore, more than half of the participants 28 (59.6%) belonged to the nursing cadre (Table 3).

**Table 3:** Health worker (*N* = 47) characteristics

Heath worker Characteristics	*N* (%)
**Age**	
< 29	24 (51.1)
30–40	18 (38.3)
> 40	5 (10.6)
**Sex**	
Female	28 (59.6)
Male	19 (40.4)
**Cadre**	
Medical doctor	1 (2.1)
Clinical officer	10 (21.3)
Nurse	28 (59.6)
Nursing assistant/Midwife	8 (17.0)

### Child acceptability of CRAFFT screening

**Fig. 1:**
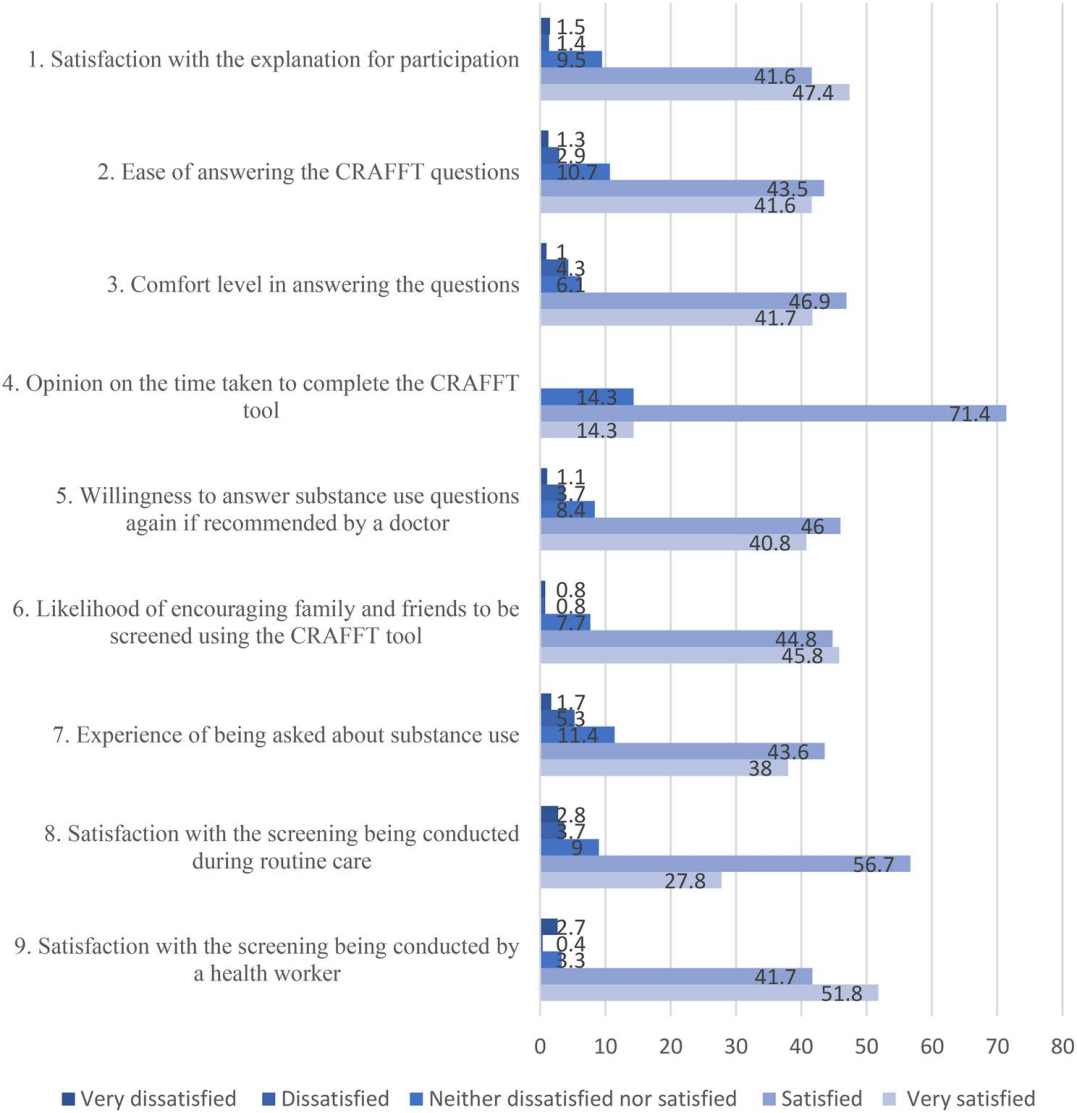
Child acceptability of CRAFFT screening displayed percentages on the Likert scale

Across all nine acceptability items, responses were predominantly skewed toward the positive end of the Likert scale (Fig. 1). For each domain, between approximately 80% and 90% of children reported being either satisfied or very satisfied, while fewer than 15% selected neutral responses and fewer than 10% reported dissatisfaction. Nearly half of the children, 394 (47.4%), were very satisfied with the explanation provided for participating in the CRAFFT screening exercise. Additionally, 41.6% found the questions in the CRAFFT tool easy to answer, and 347 (41.7%) were comfortable with the questions. Most children, 590 (71.4%), felt that the time utilized for the screening was appropriate. More than half, 419 (51.8%), were very satisfied that the screening was conducted by a health worker, and 471 (56.7%) were satisfied with the screening being integrated into their routine care.

### Caregiver acceptability of CRAFFT screening

**Fig. 2:**
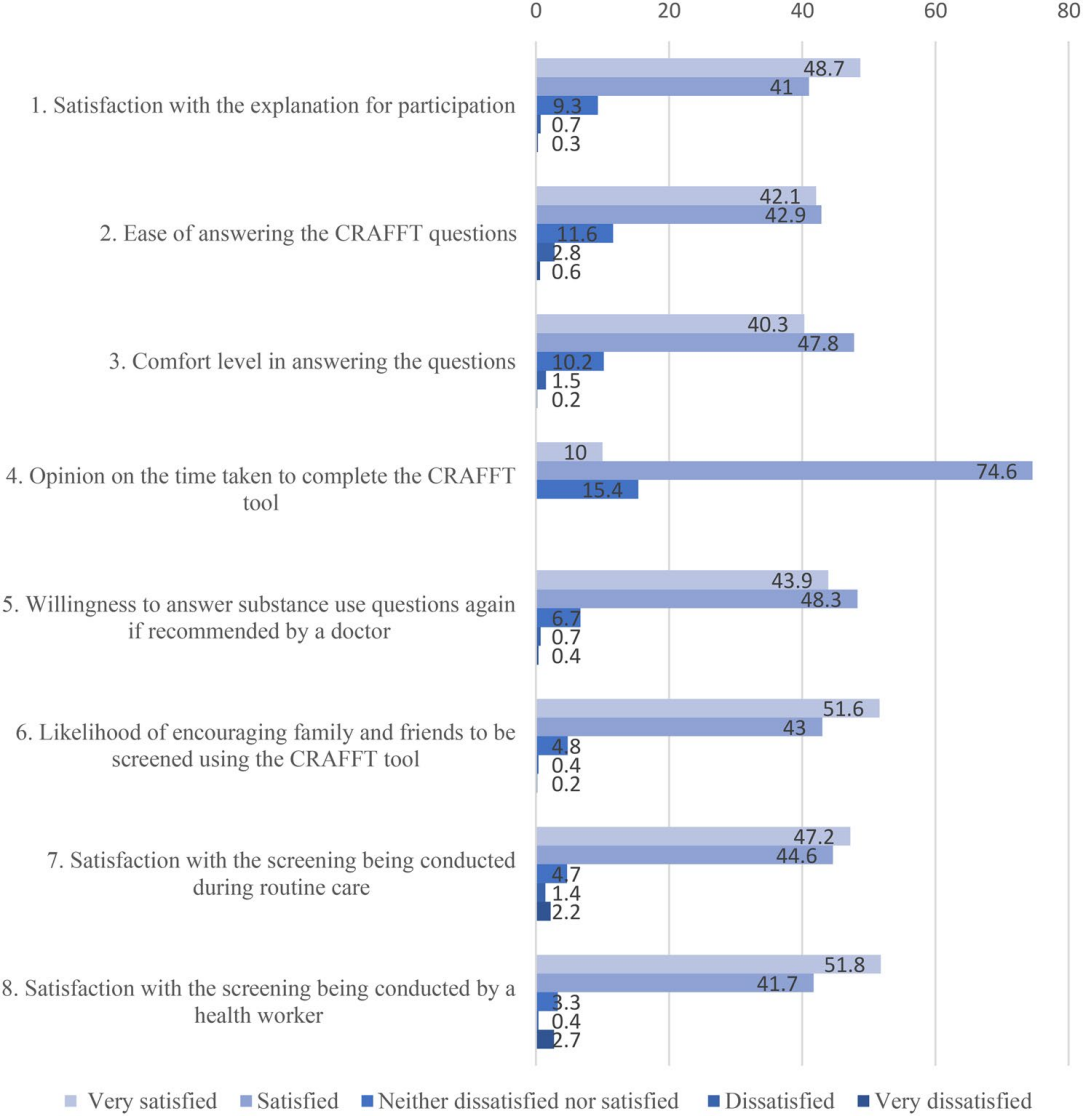
Caregiver acceptability of CRAFFT screening displayed percentages on the Likert scale

Across all eight acceptability items, caregiver responses were predominantly skewed toward acceptability. For each domain, approximately 80–90% of caregivers reported being either satisfied or very satisfied, while neutral responses were uncommon and dissatisfaction was reported by fewer than 10% of respondents (Fig. 2). Most caregivers, 602 (74.6%), felt that the time taken to administer the CRAFFT was appropriate. More than half, 418 (51.6%), were very willing to encourage their family and friends to be screened using the CRAFFT tool. Nearly half, 355 (43.9%), were very willing to have their child answer substance use questions again if recommended by a doctor. Additionally, more than half, 419 (51.8%), were very satisfied that the screening was conducted by a health worker. Several caregivers, 388 (47.8%), were comfortable with the screening questions administered.

### Health worker acceptability of CRAFFT screening

**Fig. 3:**
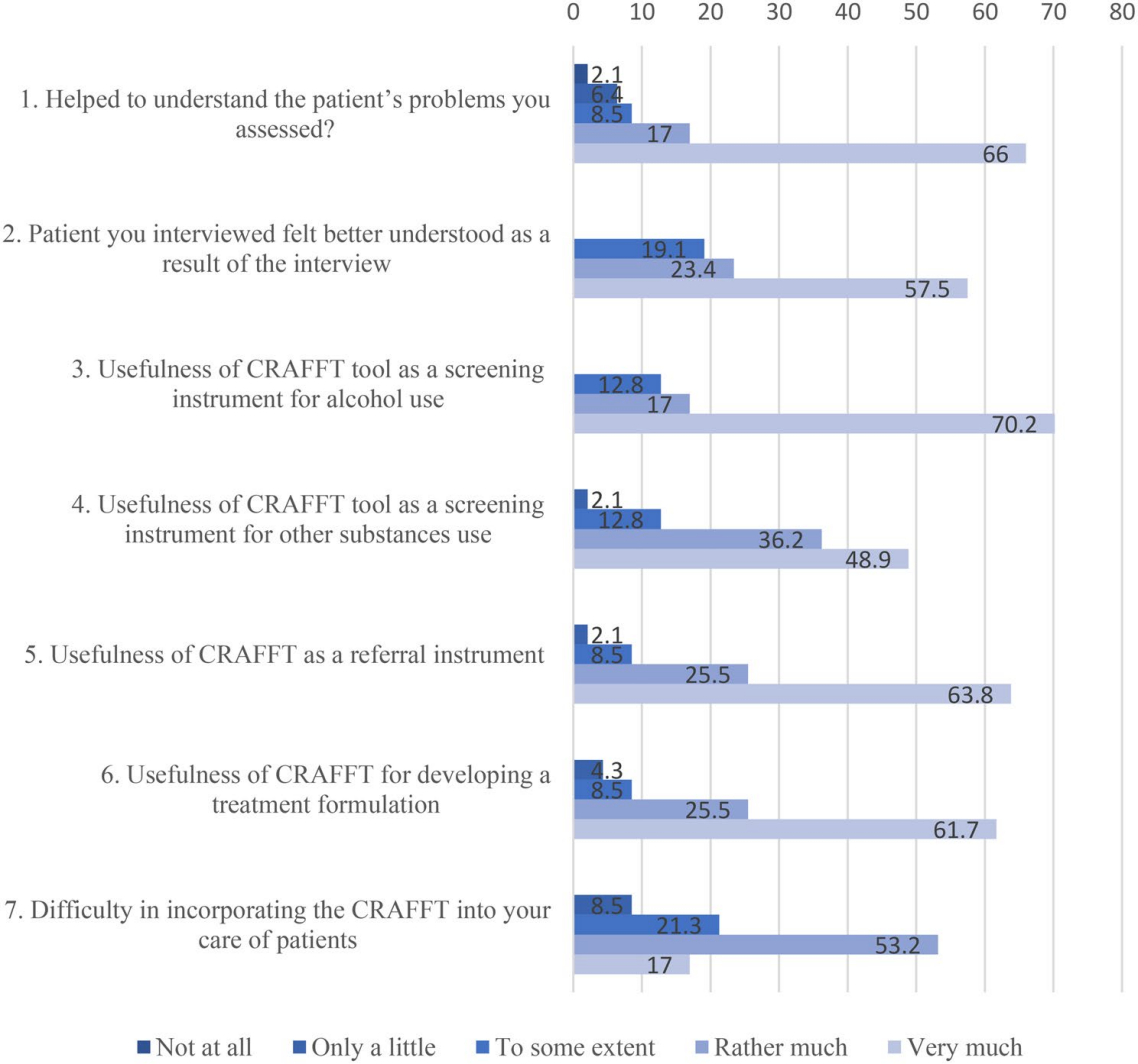
Health worker acceptability of CRAFFT screening displayed percentages on the Likert scale

Across all seven acceptability items, more than two-thirds of health workers reported that the CRAFFT tool was useful either “rather much” or “very much.” Neutral or low-endorsement responses (“not at all” or “only a little”) were uncommon across domains, indicating broadly positive perceptions of the tool’s usefulness for screening, referral, and treatment planning (Fig. 3).More than half of the health workers 31 (66.0%) felt that the CRAFFT tool greatly helped them understand the patients’ problems, 27 (57.5%) believed that the tool made their patients feel better understood 33 (70.2%), found the CRAFFT tool very useful for screening alcohol use, and 30 (63.8%) saw it as a valuable referral instrument. Additionally, many 29 (61.7%) considered it very useful for treatment formulation.

### Child and adolescent feedback about CRAFFT screening

Out of the 854 children and adolescents who participated in the study, 601 (70.1%) provided verbal feedback about their experience with the CRAFFT screening. The feedback was both positive and negative. About a third of the 179 (29.8%) of them reported having no challenges with the screening exercise. Many noted they were comfortable participating 62 (10.3%), found the tool good 58 (9.7%), considered it educative 35 (5.8%), and found it easy to answer 17 (2.8%). However, several participants noted that the screening was time-consuming 51(8.5%). Some expressed discomfort 21 (3.5%) while answering the questions, while others expressed fear 18 (3.0%) about disclosure and the possibility of being found out by their parents. Lastly, some expressed concern about confidentiality and privacy during the interview 16 (2.7%) (Table 4).

**Table 4:** Verbal feedback about CRAFFT screening from Children and adolescents (*N* = 601)

	Frequency	%
**Positive feedback**		
No challenges	179	29.7
Comfortable, happy and free to answer	62	10.3
Good tool	58	9.6
Educative tool	35	5.8
Shared experience about substance use	34	5.7
Easy to answer	17	2.8
Very good	13	2.2
Very helpful	13	2.2
Too quick	8	1.4
Willing to answer again	4	0.7
**Negative feedback**		
Time consuming/ too long	51	8.5
Discomfort answering the questions	21	3.5
Expressed fear of disclosure	18	3.0
Privacy concerns	16	2.7
Too many questions	13	2.2
Unsure about how they felt	13	2.2
Unnecessary among non-users	11	1.8
Reward for participation	11	1.8
Problems with questions (tricky, not clear, hard)	10	1.7
Sensitive questions	9	1.5
Language barrier	5	0.8
**Total**	601	100.0

Child acceptability feedback

Question: Please document patients verbal comments regarding instrument acceptability

### Caregiver feedback about CRAFFT screening

After the completion of the screening exercise for the children, all 808 caregivers were asked to provide verbal comments regarding the screening tool. Out of these, 529 (65.5%) caregivers gave feedback. Both positive and negative comments were received. Regarding positive feedback, 114 (21.6%) caregivers reported that the tool was very good for screening alcohol use in children. Many felt that the screening for alcohol and substance use should be part of regular care 59 (11.2%) and found it helpful in identifying problematic use 41 (7.8%). Some caregivers were very happy and supportive of the screening exercise 39 (7.4%), considering it an important tool for identifying those with alcohol and other substance use problems. However, some caregivers noted concerns about the screening being time-consuming 43 (8.1%). Others expressed worry 37 (7.0%) about the appropriateness of the screening exercise for different age groups, with some noting it was not suitable for younger children or for some religious groups particularly for Muslims or Pentecostal Christians whose beliefs were against consumption of alcohol (Table 5).

**Table 5:** Verbal Feedback about CRAFFT screening from Caregivers

	Frequency	%
**Positive feedback**		
Very good	114	21.6
Continue screening	59	11.2
Helpful	41	7.8
Happy and supportive of exercise	39	7.4
No challenges	33	6.2
Important tool	25	4.7
Acceptable	15	2.8
Recommend to be moved to community	13	2.5
Appreciative of the exercise	12	2.3
Teaching guide	6	1.1
Flexible, easy to understand	5	0.9
Too quick	2	0.4
**Negative feedback**		
Time consuming	43	8.1
Expressed worry about screening (Age and religion)	37	7.0
Too many questions	23	4.3
Other health concerns	19	3.6
Too long	14	2.6
Reward for participation	12	2.3
Privacy concerns	7	1.3
Shorten tool	7	1.3
Child fearful	3	0.6
**Total**	529	100

Caregiver feedback

Question: Please document caregiver’s verbal comments regarding instrument acceptability

### Health worker feedback about CRAFFT screening

A total of 47 health workers participated in the screening exercises. After completing the acceptability questions, they were also asked to provide verbal comments about the screening exercise. All 47 (100%) gave feedback. They noted that the screening tool was easy to administer 8 (17%) and effective in identifying substance use problems 8 (17%). Furthermore, 18 (38.4%) recommended that the tool be adopted in routine care, community settings, and schools. They also found it useful for managing children and adolescents with substance use issues. However, they expressed concerns about the screening exercise being time-consuming 4 (8.5%). Additionally, they noted that the health facilities’ environment did not offer adequate privacy for the screening exercise due to space limitations and the lack of designated areas for screening 4 (8.5%) (Table 6).

**Table 6:** Health worker feedback about CRAFFT screening

	Frequency	%
**Positive feedback**		
Should be adopted in routine care, community and schools	18	38.4
Easy	8	17.0
Good for screening	8	17.0
Useful	5	10.6
**Negative feedback**		
Time consuming	4	8.5
Privacy/space concerns	4	8.5
**Total**	47	100.0

Health worker feedback

Question: Document any thoughts, challenges and recommendations in regard to CRAFFT screening

## Discussion

The acceptability of substance use screening has been evaluated in various settings. However, to our knowledge, no study has assessed the acceptability of substance use screening among children using the CRAFFT tool. Our findings demonstrate that the CRAFFT screening tool is largely acceptable to children and adolescents, their caregivers, and health workers. Open-ended responses highlighted the importance of privacy for health service users and time-related challenges for health workers. These findings have public health implications for the integration of substance use screening into routine care and expanding its use in community settings and schools.

In this study, substance use screening among children and adolescents was generally acceptable, with perceptions shaped by common cross-cutting factors such as confidentiality, perceived relevance, and feasibility within routine care. Our findings align with prior studies from diverse settings showing that acceptability of screening tools is high when respondents perceive the process as understandable, respectful, and protective of privacy. For example, in a study conducted in the Democratic Republic of Congo and Ethiopia using Audio-Computer Assisted Self-Interview (ACASI), nearly 90% of adolescent girls in the DRC and approximately 75% in Ethiopia reported that screening questions were easy to understand, highlighting the role of clear communication and age-appropriate delivery in shaping acceptability [30]. Similarly, a study in Kenya among youth aged 15–24 years found peer-delivered screening to be acceptable, particularly where confidentiality and trust were emphasized [31].

Across studies, assurances of confidentiality consistently emerge as central to acceptability, especially for younger populations. In our study, concerns about privacy and fear of disclosure influenced how comfortable children and adolescents felt engaging with screening, echoing findings by Lynch et al. [32], who noted that the presence of parents or caregivers during screening can reduce adolescents’ willingness to engage openly [32]. At the same time, caregivers’ acceptance of screening was often motivated by recognition of the importance of early identification and prevention of substance use disorders. This aligns with evidence from pediatric emergency and primary care settings where most caregivers supported screening, with endorsement rates of up to 84% in some studies [33, 34]. However, caregiver acceptability is not uniform: studies such as Sandelich et al. [35] highlight persistent concerns around privacy, stigma, judgment, and legal implications, indicating that acceptability is contingent rather than absolute [35]. Health worker acceptability in our study followed a similar pattern of conditional support. Most health workers viewed the CRAFFT tool as useful and relevant for understanding patient problems, facilitating referrals, and informing care, consistent with findings from SBIRT implementation studies in pediatric and primary care settings [5, 36]. However, perceived acceptability was closely linked to feasibility considerations, particularly workload, time constraints, and availability of referral pathways. These concerns mirror those reported elsewhere, where health workers express reluctance to conduct routine screening in the absence of adequate training, institutional support, and clear follow-up options [37]. In resource-limited settings such as Uganda, these challenges are amplified by high patient volumes and limited mental health services, as also documented by Kabunga et al. [38].

The mean time required to administer the CRAFFT screening tool in this study (17–18 min) was substantially longer than what has been reported in high-income settings. For example, Harris et al. reported an average completion time of 1.6 min among adolescents aged 12–17 years in Massachusetts [39]. This marked difference reflects the importance of contextual factors in shaping the feasibility of screening tools when applied in routine primary healthcare settings, particularly in low- and middle-income countries (LMICs).

Several interrelated factors likely contributed to the longer administration time observed in this study. First, our study population included younger children aged 6–11 years, who may require additional explanation, reassurance, and time to comprehend abstract or sensitive questions related to substance use. Younger children may also have limited familiarity with clinical questioning and may be more hesitant to disclose information without adequate rapport-building, which can lengthen the screening process. Previous studies have highlighted that developmental stage significantly influences how children engage with substance use screening tools, with younger children often requiring more interactive and supportive approaches [40].

Second, the clinical context in which screening was conducted differed substantially from that of the Massachusetts study. The latter was implemented in specialized pediatric or adolescent care settings with clinicians experienced in adolescent health and with workflows designed to accommodate preventive screening. In contrast, screening in our study was embedded within routine primary healthcare facilities in Uganda, many of which operate under severe human resource constraints. Uganda’s health system faces chronic shortages of trained health workers, high patient volumes, and inequitable distribution of staff, particularly in lower-level facilities where a single health worker may be responsible for all outpatient services [41]. Although the study protocol specified that screening commenced after consent had been obtained, in practice, the realities of busy primary care settings meant that the screening encounter often included time spent establishing rapport, reassuring children about confidentiality, and clarifying the purpose of the questions. These interactions were not intended to modify the CRAFFT items themselves, but rather reflected ethical and relational processes necessary for engaging children meaningfully in these clinical environments. Similar findings have been reported in other LMIC studies, where provider-patient communication, trust-building, and contextual explanation are integral to service delivery but are rarely accounted for in estimates of screening duration [16, 20].

Importantly, the extended duration observed in this study highlights a critical implementation gap between theoretically brief screening tools and the realities of delivering them to children in routine care settings. Implementation science literature has emphasized that feasibility is shaped not only by tool characteristics but also by workflow integration, staffing levels, and patient population needs (Damschroder et al. 2009). Our findings suggest that without adaptation such as task-shifting, simplified administration procedures, or alternative delivery models (e.g. assisted self-administration or community-based screening) routine CRAFFT screening may impose a substantial time burden on already overstretched primary care providers.

These findings have important implications for scale-up as well. While screening tools like CRAFFT may be psychometrically sound and acceptable in principle, their successful integration into LMIC primary care settings will require explicit consideration of staffing constraints, age-appropriate communication strategies, and health worker workload. Future implementation efforts should explore streamlined workflows, additional training focused on child-centered communication, and health system supports that enable screening to be conducted efficiently without compromising ethical standards or care quality.

### Perceptions about the substance use screening

Participants’ perceptions of the CRAFFT screening tool were largely positive, with many describing it as educative, easy to understand, and useful in identifying risk behaviors among children and adolescents. These findings align with existing literature on the acceptability of the CRAFFT tool in various settings. For instance, studies by [42, 43] have similarly reported high levels of acceptability among both adolescents and healthcare providers, particularly highlighting the tool’s simplicity and relevance for early detection of substance use.

Despite overall positive feedback, participants in this study identified several barriers to the effective implementation of CRAFFT screening. Reported challenges included lengthy screening times, privacy concerns, the sensitivity of the questions, and issues related to age and cultural or religious appropriateness. These concerns mirror those documented in studies from high-income settings. For example, [44] found that time constraints and the complexities of parental involvement were significant barriers to implementing substance use screening in pediatric care. Similarly, Harris et al. [5] identified a lack of provider training and skepticism about the efficacy of brief interventions as critical implementation challenges [5].

These barriers have important public health implications. Lengthy screening procedures and lack of privacy may discourage children and caregivers from engaging openly with screening tools, thereby limiting early identification of risky substance use behaviors. Moreover, the sensitivity of some CRAFFT questions may evoke discomfort or perceived judgment, particularly in conservative or highly religious communities. This is supported by Gryczynski et al. [45], who reported that both adolescents and providers may feel uneasy when discussing certain substance use behaviors, especially in settings lacking confidentiality or proper training [45].

Addressing these challenges requires a multipronged approach. First, streamlining the screening process by integrating CRAFFT into routine clinical workflows could help reduce time burden. Second, ensuring that questions are framed in a developmentally appropriate and culturally sensitive manner may increase acceptability. Third, reinforcing confidentiality and safe disclosure especially during one-on-one interviews without caregivers present may enhance trust and openness during screening [36].

Additionally, participants suggested that CRAFFT screening should be expanded beyond health facilities to include community and school-based settings. This recommendation is supported by findings from Harris et al. (2016), who argue that integrating screening into schools and youth programs increases accessibility and reduces stigma by normalizing substance use discussions as part of broader adolescent health promotion [46].

In conclusion, while the CRAFFT screening tool is generally well accepted, its implementation is influenced by contextual factors such as time, privacy, and cultural appropriateness. Addressing these barriers and expanding the screening environment can significantly enhance uptake and effectiveness, ultimately contributing to improved early detection and intervention for substance use among children and adolescents.

### Study limitations

This study has several limitations that should be acknowledged. First, the CRAFFT screening was conducted in outpatient settings that may not have ensured sufficient privacy. This lack of confidentiality could have influenced participants’ willingness to respond honestly, thereby affecting the observed acceptability of the screening and potentially introducing information bias. It is plausible that conducting the screening in more private settings might have yielded different results.

Second, social desirability bias may have affected responses, particularly because interviews were conducted by health workers based in health facilities located near participants’ communities. Participants may have been inclined to provide responses they believed were expected or socially acceptable, especially when interviewed by familiar healthcare personnel.

Third, language barriers may have been present, especially in larger facilities like the Regional Referral Hospital, which serves diverse populations from multiple districts. Not all health workers in such settings may have been proficient in the local languages spoken by participants, potentially hindering clear communication and affecting comprehension of the screening questions. Fourth, the study may have been subject to central tendency bias, whereby respondents tend to avoid extreme response options on Likert scales and instead select neutral or middle-range categories. This bias is particularly plausible in our context, where children, caregivers, and health workers were interviewed within health facility settings and may have felt hesitant to express very strong positive or negative opinions. Younger participants and caregivers may have been cautious about appearing overly critical of health workers or the health system, while health workers themselves may have avoided extreme responses due to professional norms or perceived expectations. As a result, some responses may have clustered around mid-scale categories, potentially masking stronger underlying perceptions and leading to either underestimation or overestimation of true acceptability. Additionally, the study did not capture data on health workers perceptions about the duration it took to administer the CRAFFT tool. This omission limits a fuller understanding of the time burden of the screening process, which is an important dimension in assessing feasibility and acceptability in routine clinical practice.

Despite these limitations, this study is the first of its kind in Uganda to assess the acceptability of CRAFFT screening among children, caregivers, and health workers. The inclusion of both structured Likert-scale questions and open-ended items enriched the findings by offering deeper insights into participants’ perceptions, perceived barriers, and suggestions for improving screening practices.

This study also applied six of the seven constructs from the Theoretical Framework of Acceptability (TFA) to guide questionnaire development. These included affective attitude, burden, ethicality, intervention coherence, perceived effectiveness, and self-efficacy. Each construct was operationalized through targeted Likert-scale items assessing emotional responses, perceived effort, alignment with values, clarity of the tool’s purpose, confidence in engagement, and perceived usefulness of the screening.

However, the construct of opportunity costs which captures the extent to which participants perceive the need to sacrifice something valuable (e.g. time, emotional comfort, or privacy) was not explicitly measured. We recognize this as a limitation. Future iterations of the tool should incorporate items to evaluate opportunity costs in order to more fully assess acceptability in accordance with the complete TFA model.

## Conclusions

This study provides novel evidence that CRAFFT screening is acceptable among children and adolescents aged 6–17, their caregivers, and primary healthcare workers in a Ugandan setting. While acceptability is high, implementation is influenced by time constraints, health system limitations, confidentiality concerns, and sociocultural context. Addressing these challenges through training, system integration, and expansion into community and school settings is critical. Ultimately, improving the acceptability and feasibility of substance use screening can enhance early detection and intervention, contributing to improved adolescent health outcomes in low-resource settings.

## Data Availability

The datasets generated and/or analysed during the current study are available upon request.

## Abbreviations

AUD: Alcohol Use disorders
LMICs: Low- and Middle-Income Countries
SARA: Service Availability and Readiness Assessment
SU: Substance Use
SUD: Substance Use disorders
WHO: World Health Organization
